# Quantification of the Survival Disadvantage Associated with Major Amputation in Patients with Peripheral Arterial Disease

**DOI:** 10.3390/jcm14010104

**Published:** 2024-12-27

**Authors:** Maria Elisabeth Leinweber, Emanuel Greistorfer, Julia Rettig, Fadi Taher, Miriam Kliewer, Afshin Assadian, Amun Georg Hofmann

**Affiliations:** Department of Vascular and Endovascular Surgery, KliniK Ottakring, Montleartstrasse 37, 1160 Vienna, Austria

**Keywords:** major amputation, peripheral artery disease, lower extremity, mortality, risk factors

## Abstract

**Objective**: Despite advancements in vascular surgery, the mortality among peripheral arterial disease (PAD) patients undergoing major amputations remains high. While a large body of evidence has previously covered survival rates after major amputation, there is less evidence regarding the associated survival penalty from an epidemiological perspective. The present analysis aimed at quantifying the survival disadvantage after major lower limb amputation while investigating which factors are associated with mortality in this patient cohort. **Methods**: Data from 246 PAD patients undergoing major amputations were retrospectively collected and matched with mortality records from the Austrian National Death Registry. Life expectancy was estimated using population-based life tables, and differences between observed and expected survival were analyzed across subgroups. **Results**: The median follow-up was 492 days (Q1–Q3: 73–1438), and 82.5% (n = 203) of patients died, with cardiovascular events being the leading cause (41%). A profound discrepancy between estimated (4697 days, Q1–Q3: 2962–6236) and observed survival (457 days, Q1–Q3: 73–1438, *p* < 0.001) was seen. In men, an associated median survival penalty of 11.2 years was observed, equivalent to a proportionate reduction in life expectancy of over 90%, while the difference in women was 8.7 years, equaling a reduction of 84.6%. In a multiple regression model, 1 year in life expectancy was associated with a survival penalty of −0.96 years, thereby affecting younger patients with the highest life expectancies the most. **Conclusions**: Major amputation in PAD patients is associated with a significant reduction in survival compared to standardized mortality rates in the general population. The survival disadvantage exceeds 70% of estimated survival times in over 70% of patients. Elevated mortality rates after major amputation in PAD patients should not be interpreted as a causal relationship but as a surrogate for impaired systemic cardiovascular health.

## 1. Introduction

Chronic wounds and tissue loss arising from conditions such as peripheral arterial disease (PAD), diabetic angiopathy, and chronic venous insufficiency represent the leading causes of amputations globally [1,2,3]. Major risk factors associated with the pathogenesis of PAD include smoking, arterial hypertension, dyslipidemia, or diabetes [4,5]. The highest prevalence and incidence rates are still observed in North America, followed by European countries. However, especially East and South-East Asian countries have seen relevant increases in both metrics over the previous two decades [5]. Over 200 million individuals are estimated to be affected by PAD, and amputations due to vasculopathy continue to be a prevalent surgical intervention in many regions worldwide [6]. The recently published European Society of Cardiology guidelines on the management of peripheral arterial and aortic disease state that about 1% of patients with intermittent claudication will subsequently undergo major lower extremity amputation and at least 9% of patients with chronic limb-threatening ischemia. It is additionally recommended that the individual amputation risk in PAD patients with chronic wounds should be estimated using the Wound, Ischemia, and foot Infection (WIfI) classification [7].

Major amputations are associated not only with severe physical, psychosocial, and economic consequences but also with a notably high mortality [8,9,10]. In an international analysis of health insurance claims data, documented hospital mortality rates following major amputation ranged from 6.1% in Finland to 20.8% in Hungary, with Austria reporting a rate of 14.3% [2]. In a meta-analysis of 16 studies, mortality rates of 47.9% at one year and 70.6% at three years among major amputees were reported [11]. While major amputation may not directly cause increased mortality but rather serve as a surrogate marker for an overall impaired cardiovascular status, the paucity of studies, high heterogeneity, and lack of reporting standards make it difficult to draw definitive conclusions regarding the factors that significantly influence the survival of amputee patients [8]. Furthermore, while it is generally acknowledged that amputee patients face increased mortality compared to their peers and the general population, data quantifying this specific survival penalty remains sparse.

This retrospective study, utilizing data from patient medical records as well as population-based data from the National Death Registry and general and balanced life tables, seeks to quantify the survival penalty following major amputation in PAD patients and to identify factors associated with the increased mortality rates frequently reported in this patient cohort. In contrast to previous research focusing on mortality rates after major amputation, the present approach enables a direct investigation of the survival disadvantage within this patient cohort compared to the general population.

## 2. Methods

### 2.1. Study Design and Data Collection

This study was conducted as a retrospective single-center analysis. A prospective protocol was developed. Patient data, including demographics, comorbidities, and treatment characteristics, were extracted from medical records maintained at the study center. The study included all patients aged over 18 years who underwent major amputation due to compromised micro- or macroangiopathic vascular conditions between 2012 and 2016. This time period was specifically chosen in order to allow long-term follow-ups as well as mitigate the effects of the COVID-19 pandemic on mortality rates that could potentially interfere with the analyses and results. Patients who had undergone a major amputation because of trauma or an oncological diagnosis were excluded from the study. In summary, 246 patients met the inclusion criteria, follow-up data were available for 227 patients.

Data regarding comorbidities were obtained from the electronic health records based on ICD-10 classification. Mortality data were obtained from the Austrian National Death Registry to ensure comprehensive and accurate tracking of patient outcomes. Plausibility checks were conducted on the collected data to ensure accuracy and consistency. All patient data were anonymized, stored securely, and accessed only by authorized personnel involved in the study.

### 2.2. Estimation of Life Expectancy

The estimated life expectancy for each patient was calculated using data derived from general and balanced life tables provided by the National Statistical Office (Statistik Austria). These life tables allowed for a standardized estimation of expected survival based on population-level data, which was then used to assess the survival penalty associated with major amputation. Estimated life expectancies for each patient were calculated after fitting spline functions for both male and female patients separately based on the standardized life tables. Patient age was subsequently used as an input for the respective spline function.

### 2.3. Analysis

All statistical analyses were conducted using R (v. 4.1.3, R Foundation for Statistical Computing, Vienna, Austria). Descriptive statistics were used to display study population characteristics. Categorical variables are shown as frequencies and proportions. Continuous variables are shown as the median and Q1–Q3. This analysis followed a three-step approach: First, a crude survival probability was estimated using Kaplan–Meier curves and stratified by various subgroups, utilizing data from the national death registry for each patient in the dataset. Next, an estimated life expectancy for each patient was calculated based on population-based general and balanced life tables, with the day of the operation as the index date. Estimated and observed life expectancy were compared across different covariates. In the third step, a linear regression model adjusting for various covariates was derived.

For standard hypothesis tests, a *p* value < 0.05 was considered statistically significant, whereas, for predictors in the regression model, a *p*-value < 0.1 was considered the threshold for elimination in the models.

## 3. Results

### 3.1. Patient Demographics

Overall, 246 patients with PAD underwent major amputation between 2012 and 2016, with 70.8% undergoing transtibial amputation and 29.2% undergoing transfemoral amputation. Most patients were male (65.6%), with a median age of 72 years at the time of surgery (Q1–Q3: 65–79 years). At the time of hospital admission, 17% of patients were diagnosed with critical limb ischemia at Fontaine stage III, while 83% presented with Fontaine stage IV, involving minor or major tissue loss in the lower limbs. A total of 25 patients had already undergone major amputation on the contralateral leg. Patient characteristics are summarized in Table 1.

### 3.2. Treatment Characteristics and Postoperative Complications

Most patients underwent surgery under general anesthesia (74.5%), while 25.5% were operated on under regional anesthesia. Postoperative minor and major complications were documented in 127 patients. Stump complications like wound infections, hematomas, and tissue necrosis were the most common, occurring in 43.5% of all patients. Pulmonary complications were observed in 16 patients (6.5%), 13 patients (5.3%) developed sepsis, and 26 patients (10.6%) experienced a cardiac complication (cardiac decompensation, exacerbation of congestive heart failure, myocardial infarction, or cardiac arrhythmia). Overall, 27.6% required surgical reintervention due to stump complications. The median length of hospital stay was 55 days (Q1–Q3: 34–87). For patients with complications, it was 71 days (Q1–Q3: 44–101), and it was 44 days (Q1–Q3: 29–60) for those with a complication-free course (*p*-value < 0.001).

### 3.3. Survival Times

The median follow-up duration was 492 days. During the follow-up period, 203 patients died, contributing to a median survival of 457 days (Q1–Q3: 73–1438) for the entire cohort. The median length of follow-up for patients without a mortality event (n = 24) was 1869 days (Q1–Q3: 494–2778). The cause of death was documented for 200 patients. Most patients (41%) died due to cardiovascular events such as myocardial infarction, cardiac decompensation, or cardiac arrhythmia. Additionally, 1.5% of patients died from a stroke, 7.5% died because of a malignant disease, and 10.0% died from pulmonary events, including pneumonia or exacerbation of known chronic obstructive pulmonary disease. Only 6.5% died due to stump-related complications, infections, or sepsis.

Women (median survival: 615 days, Q1–Q3: 82–1462) had a higher observed median survival compared to men (median survival: 410 days, Q1–Q3: 71–1359), though this difference was not statistically significant (*p*-value: 0.95).

**Figure 1:**
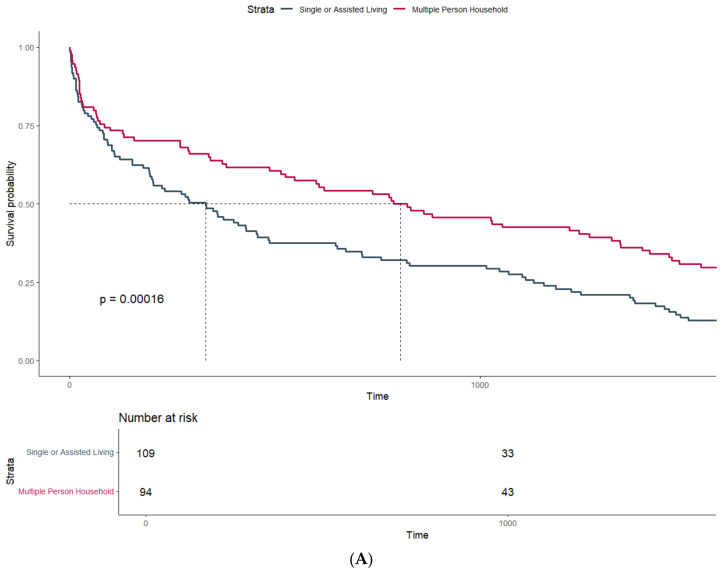

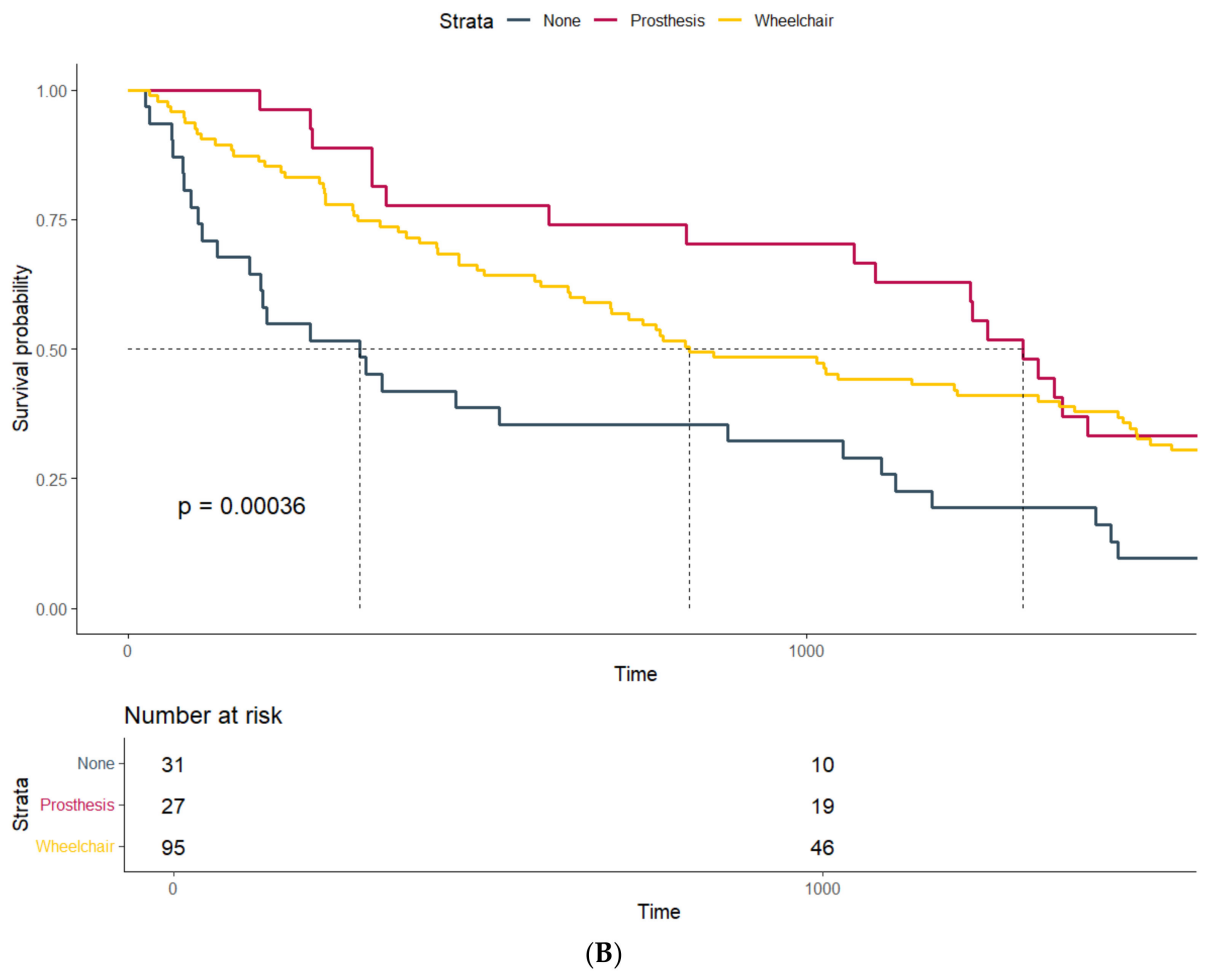
Survival probability estimated by Kaplan–Meier curves and stratified by (**A**) household size and (**B**) mobility status at discharge.

Patients undergoing transtibial amputation (738 days, Q1–Q3: 102–1507) had significantly higher median survival than patients with transfemoral amputations (247 days, Q1–Q3: 29–738, *p*-value: 0.009). Additionally, there was strong evidence for a higher median survival in patients living in a multi-person household, with 823 days (95%CI: 487–1460) compared to 344 days (95%CI: 202–515) in patients living alone (Figure 1A) (*p*-value: < 0.001).

The survival of patients stratified by their mobility at discharge also differed significantly (*p*-value: <0.001): Patients with a prosthetic fitting had a median survival of 1320 days (Q1–Q3: 722–1819) compared to a median survival of 828 days (Q1–Q3: 356–1736) in patients dependent on a wheelchair after amputation. Patients confined to bed after surgery showed the lowest median survival, with only 342 days (Q1–Q3: 106–1121) (Figure 1B). However, this association might be confounded by the lower age of patients suited for prosthetic fitting and wheelchair users compared to those confined to bed after amputation (72.3 years vs. 72.1 years vs. 84.1 years).

### 3.4. Survival Penalty Associated with Major Amputation

**Figure 2:**
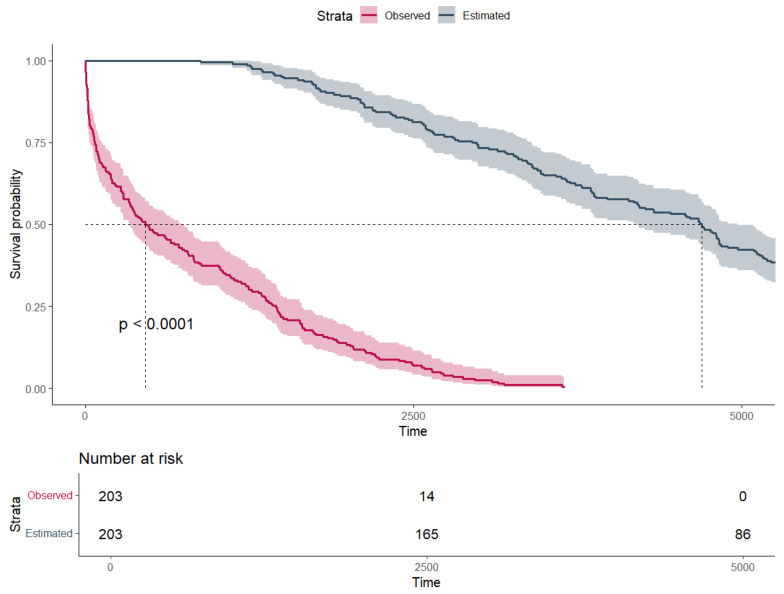
Survival probability estimated by Kaplan–Meier curves and stratified by estimated and observed survival (time in days).

**Figure 3:**
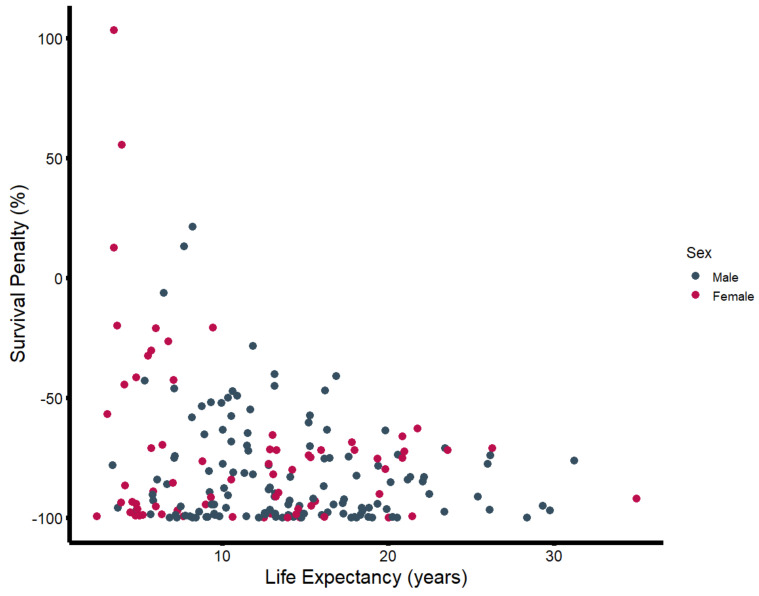
Survival penalty (in percent) plotted against estimated life expectancy (in years), stratified by sex.

In the second step, the estimated life expectancy was calculated to determine the median survival penalty associated with major amputation. There was strong evidence supporting a significant difference between observed and estimated survival, with an observed median survival of 457 days (Q1–Q3: 73–1438) vs. an expected median survival of 4697 days (Q1–Q3: 2962–6236) (*p*-value: < 0.001) (Figure 2). As seen in Figure 3, the majority of patients (72.8%) undergoing major amputation lost more than 70% of their estimated life expectancy after surgery. Of the 24 survivors, 50% exhausted less than 23% of their estimated survival time (Q1–Q3: 7.6–34.2%).

As displayed in Table 2, the median difference between observed and estimated life expectancy differed across various subgroups. Male patients had a median survival disadvantage of 11.2 years of estimated life expectancy, equivalent to a proportionate reduction in life expectancy of over 90%, while the difference in women was 8.7 years, equaling a reduction of 84.6%. Patients eligible for prosthetic fitting showed a median difference between estimated and observed life expectancy of −11.4 years (−74.7%) compared to −10.7 years (−79.8%) in wheelchair users and −5.7 years (−85.9%) in patients confined to bed.

**Figure 4:**
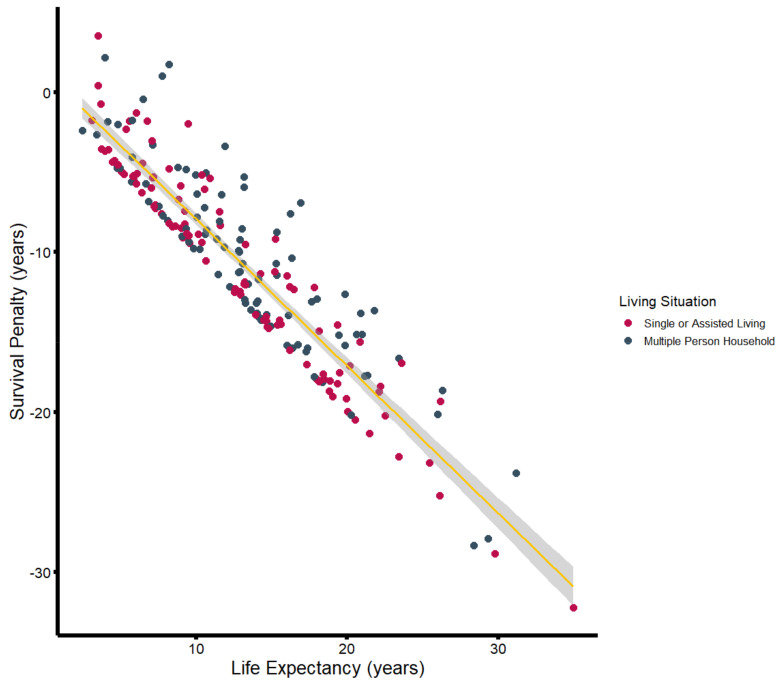
Survival penalty (in years) plotted against estimated life expectancy (in years), stratified by household size. The yellow line illustrates the associated linear regression including the 95%CI (gray-shaded area).

Substantial discrepancies were also observed when patients were stratified by household size (Table 2; Figure 4). Patients living in a multi-person household (n = 94) had a median reduction of 9.9 years (−81.5%) in life expectancy, while solitary-living patients (n = 50) exhibited a median difference between observed and estimated life expectancy of more than 12.1 years (−91.6%). Patients who lived alone but received support from either relatives or outpatient care services (n = 18) showed a median reduction of 12.0 years (−90.8%). Patients living in nursing homes had a median reduction of 5.4 years (−92.4%). While patients living alone and those in multi-person households were of similar age (multi-person household: 72.6 years; living alone: 72.6 years; living alone with support: 74.4 years), patients living in nursing homes were significantly older, with a median age of 83.2 years.

In the third step, a linear regression model was developed, adjusting for demographics, comorbidities, and social anamneses using stepwise variable selection. The final model is shown in Table 3. Proceeding from an estimated baseline survival (benefit) of 4.03 years (95%CI: 1.68, 6.38), patients exhibit a decreasing life expectancy with an increasing ASA score (r = −0.86, 95% CI: −1.49, −0.23), whereas living in a multi-person household had a protective effect (r = 1.19, 95% CI: 0.52, 1.85). A strong negative correlation was observed between estimated life expectancy and observed survival penalty (r = −0.923, 95%CI: −0.941, −0.900). In a multiple linear regression model, each additional year in life expectancy is associated with a loss of −0.96 years (95%CI: −0.97, −0.87). Considering the narrow confidence interval, there is strong evidence that the survival penalty experienced after major amputation is a relative effect associated with survival estimates, affecting younger patients with the highest life expectancies the most.

## 4. Discussion

Peripheral artery disease and consecutive amputations represent a relevant health burden in Western countries. Mortality risk assessments remain a matter of big interest in PAD patients [12,13,14]. While mortality rates after many vascular surgeries have decreased over the past decades, mortality following major amputations remains high [11,15,16]. For a considerable time, major amputation was often viewed as the irreversible last resort in cases of failed revascularization [8]. However, in recent years, there has been a growing trend in publishing data on major amputation as evidenced by 429 PubMed indexed items in 2013 using the search term “major amputation” compared to 839 in 2023 [17]. Despite the increasing body of evidence regarding the high mortality associated with major amputation, questions remain about the extent of the reduction in life expectancy [18,19].

Therefore, we conducted a three-step analysis using single-center data from medical patient records as well as data from the National Death Registry and general and balanced life tables of the National Statistical Office.

This analysis revealed a significant difference in the estimated and observed life expectancy among PAD patients undergoing major amputation. Most patients experienced a severe reduction in their survival compared to estimated life expectancies derived from the general population, with more than a 70% difference between estimates and observations in most of the cases. As a matter of fact, the survival penalty experienced by patients after having undergone lower limb amputations is foremost a relative penalty. On average, survival time after major amputation is 70% shorter than the estimated survival time in patients with a documented mortality event, which translates to a greater absolute loss in younger patients, highlighting the particularly devastating prospects for patients requiring a major amputation due to PAD at an earlier age.

In 24 survivors (10.6%), 75% exhausted less than 35% of their estimated survival at the last recorded follow-up. While their exclusion can theoretically inflate the quantified survival disadvantage minimally, they were still within the observed survival periods of deceased patients. It is, therefore, reasonable to assume that they might be subjected to a similar survival penalty as patients with recorded mortality events.

When stratified by demographic factors, comorbidities, and social characteristics, the data revealed that certain subgroups were disproportionately affected. Male patients, those who were confined to bed, and solitary living individuals exhibited the most significant reductions in life expectancy. That social isolation and loneliness are relevant risk factors for physical, cardiovascular, and mental health has been shown in previous studies, and more awareness regarding this topic has been raised in recent years [20]. For instance, loneliness has been shown to be associated with chronic heart failure [21,22], coronary artery disease [23], and several cardiovascular risk factors [24]. Mechanisms of this phenomenon have been investigated, and worse health behaviors, poorer sleep quality, and vital exhaustion have been hypothesized [20,25].

Additionally, the mitigated survival disadvantage observed in patients who were eligible for prosthetic fitting suggests that functional mobility is associated with long-term outcomes. Several previous studies have suggested that prosthetic fitting might be associated with a decreased likelihood of mortality [26,27,28]. Postoperative mobility and the ability to participate in social, work, and recreational activities have a strong impact on the quality of life after amputation [29,30,31]. Whether the link between prosthetic fitting and higher survival is more associative than causal is not conclusively investigated. Non-suitability for prosthetic fitting might be a surrogate for overall poorer health status, unfavorable body composition (overweight/underweight), and/or higher patient age. The association between non-ambulation and increased mortality has been demonstrated not only in amputees but also in patients undergoing revascularization [32]. Therefore, mortality might not be directly associated with amputees not using prosthetics but rather with patients who are not ambulatory, thereby reflecting relevant confounding by overall health status [16]. Nevertheless, the recently published Enhanced Recovery After Surgery Society and Society of Vascular Surgery guidelines on perioperative care in lower extremity major limb amputation also specifically recommend early mobilization and acute rehabilitation to improve both physical and mental outcomes [33].

It remains to be investigated whether major amputation directly accelerates mortality after surgery, as current evidence is primarily derived from observational studies [34]. Several factors have been proposed to explain the observed increase in mortality, including the significant stress on the cardiovascular system induced by the operation, subsequent infections, wound complications, reduced mobility and quality of life, and greater metabolic demand during walking [35,36,37,38]. However, the current consensus is that major amputations may not be the direct cause of postoperative mortality but rather serve as a relevant surrogate marker for severe cardiovascular impairment and advanced disease stage. Our findings align with other analyses, which suggest that the mortality of amputees is more closely linked to associated impaired systemic cardiovascular health rather than the amputation itself [39]. This perspective is further supported by the generally high mortality observed in patients with leg ulceration and those undergoing even minor amputations. For instance, a meta-analysis has shown that the five-year mortality rate after minor amputation exceeds 44% [40]. Patients with leg ulcerations or critical limb ischemia without amputation still face a five-year mortality rate of nearly 40% [41,42].

Rather than viewing amputation as a final failure, it should be recognized as a complex and multifaceted procedure that demands comprehensive preoperative planning, diligent postoperative care, and sustained long-term support. This approach is particularly crucial given future projections, such as those for the United States, where the number of individuals living with limb loss is anticipated to double by the year 2050, potentially reaching 3.6 million patients [43]. The American Heart Association has, therefore, called for action, releasing a policy recommendation to decrease nontraumatic amputations by more than 20% by 2030 through the establishment of comprehensive preventive services, increased patient awareness of PAD, and the introduction of quality and performance measures [44]. However, it remains to be seen whether or how reducing amputation rates by optimizing revascularization strategies impacts the survival of PAD patients or whether the systemic effects of progressed atherosclerotic disease affect mortality hazards irrespective of local treatment.

The conducted methodological approach offers an alternative route to investigations that focus on mortality rates within patient cohorts after major amputation or PAD. While a significant cumulative body of evidence has so far shown that these patients are subject to limited survival rates, less attention has been raised to the quantification of this survival disadvantage. Further research should be directed at investigating mortality within these patient cohorts in reference to the general population in order to enhance our understanding in an epidemiological context.

### Limitations

The retrospective design of this analysis inherently limits the ability to infer causality and may introduce selection bias. Our data were derived from a single center, which may affect the generalizability of the results to other settings or populations. While this study aimed to quantify the survival penalty following major amputation, it lacked detailed information on the quality of life of patients. Quality of life is a crucial aspect of patient outcomes that encompasses functional, social, and psychological dimensions, all of which can significantly influence rehabilitation success and survival. Additionally, our study did not include data on subjective experiences of social isolation or loneliness, which could be significant factors influencing survival outcomes. Although marital status and household composition were recorded, these variables may not fully represent the emotional and social support that can impact long-term survival. A further limitation is related to the estimation of life expectancy using general and balanced life tables. While these tables provide a standardized method for comparison, they may not fully account for individual variations in health status or comorbidities. Consequently, the estimated life expectancy might not precisely reflect the survival expectations of our specific patient cohort. Finally, the analysis of survival penalties based on prosthetic fitting and mobility status raises the question of potential confounding factors. Patients eligible for prosthetic fitting might have better overall health and functional status, which could skew the observed association between prosthetic use and survival.

## 5. Conclusions

We found strong evidence for a significant reduction in survival times after major lower limb amputation compared to standardized mortality rates in the general population. The survival penalty exceeds 70% of the estimated survival time in over 70% of the study population with recorded mortality events, reflecting a consistent relative effect translating to higher disadvantages in younger patients. While this observation might be confounded by overall impaired health in these patients, the quantification of the survival disadvantage associated with major amputation reiterates the importance of preventing progressed stages of atherosclerotic disease. Major amputations may not be the ultimate failure of surgical revascularization but rather the failure of a comprehensive approach to atherosclerotic disease that begins with primary prevention.

## Figures and Tables

**Table 1:** Patient characteristics.

Parameter	Non-Deceased Patients (n = 43) n (%)	Deceased Patients (n = 203) n (%)	Total (n = 246) n (%)
Age	65 (57–70)	74 (67–80)	72 (65–79)
Body Mass Index (BMI)	26.2 (22.5–29.3)	24.5 (22.2–27.7)	24.7 (22.2–27.7)
Male: Female	30 (69.7): 13 (30.3)	131 (64.5): 72 (35.5)	161 (65.4): 85 (34.6)
Arterial hypertension	27 (62.8)	177 (87.2)	204 (82.9)
Coronary artery disease Previous myocardial infarction	15 (34.9) 8 (18.6)	86 (42.4) 36 (17.7)	101 (41.1) 44 (17.9)
Diabetes mellitus	21 (48.8)	128 (63.1)	149 (60.6)
History of stroke	7 (16.3)	40 (19.7)	47 (19.1)
Hyperlipidemia	20 (46.5)	74 (36.5)	94 (38.2)
Pulmonary comorbidities	13 (30.2)	64 (31.5)	77 (31.3)
Chronic kidney disease	19 (44.2%)	109 (53.7%)	128 (52.0)
Previous ipsilateral revascularization	16 (37.2%)	83 (40.9%)	99 (40.2%)
Smoking Current History of smoking Non-smokers Missing: 35 (14.2%)	27 (62.8) 4 (9.3) 8 (18.6)	79 (38.9) 15 (7.4) 78 (38.4)	106 (43.1) 19 (7.7) 86 (34.9)
ASA Score 2 3 4 Missing: 16 (6.5%)	3 (6.9) 33 (76.7) 4 (9.3)	9 (4.4) 122 (60.1) 59 (29.1)	12 (4.9) 155 (63.0) 63 (25.6)
Level of amputation Transtibial Transfemoral	33 (76.7) 10 (23.3)	141 (69.5) 62 (30.5)	174 (70.7) 72 (29.3)

**Table 2:** Median difference of observed and estimated life expectancy, stratified by patient and treatment characteristics.

Parameter	Median Difference Observed and Estimated Life Expectancy (in Years)	Median Difference Observed and Estimated Life Expectancy (in Percent)
Sex		
Male	−11.2	−90.5%
Female	−8.7	−84.6%
ASA Score		
2	−8.9	−83.9%
3	−10.2	−84.9%
4	−7.1	−96.4%
Smoking history		
Never	−8.5	−87.5%
Ex-smoker	−12.6	−87.7%
Current smoker	−12.9	−90.4%
Coronary artery disease		
Yes	−11.0	−91.3%
No	−9.7	−88.3%
Diabetes mellitus		
Yes	−10.5	−84.4%
No	−9.1	−93.4%
Household size		
Multi-person household	−9.9	−81.5%
Single living	−12.1	−91.6%
Assisted single living	−12.0	−90.8%
Nursing home	−5.4	−92.4%
Fontaine stage		
III	−8.9	−94.4%
IV	−10.0	−87.2%
Level of amputation		
Transtibial	−10.6	−84.9%
Transfemoral	−8.9	−94.3%
Mobility after discharge		
Prosthetic fitting	−11.4	−74.7%
Wheelchair	−10.7	−79.8%
Confined to bed	−5.7	−85.9%

**Table 3:** Final model of the multiple linear regression analysis. A total of 13 observations were deleted due to missingness.

Variable	Regression Coefficients from Multiple Linear Regression Model			R^2^	Adjusted R^2^
	Estimate	95% CI	*p*-Value		
Baseline	4.03	1.68, 6.38	<0.001		
Life Expectancy	−0.96	−0.941, −0.900	<0.001		
Living in a multi-person household	1.19	0.52, 1.85	<0.001		
ASA Score	−0.86	−1.49, −0.23	0.007	0.867	0.865

## Data Availability

Data can be made available upon reasonable request.
